# DNA Methylation Adjusts the Specificity of Memories Depending on the Learning Context and Promotes Relearning in Honeybees

**DOI:** 10.3389/fnmol.2016.00082

**Published:** 2016-09-12

**Authors:** Stephanie D. Biergans, Charles Claudianos, Judith Reinhard, C. G. Galizia

**Affiliations:** ^1^Queensland Brain Institute, University of Queensland, BrisbaneQLD, Australia; ^2^Neurobiologie, Universität KonstanzKonstanz, Germany; ^3^Monash Institute of Cognitive and Clinical Neuroscience, Faculty of Biomedical and Psychological Sciences, Monash University, MelbourneVIC, Australia

**Keywords:** DNA methylation, DNA methyltransferases, memory formation, memory specificity, relearning, epigenetics, honey bee, generalization

## Abstract

The activity of the epigenetic writers DNA methyltransferases (Dnmts) after olfactory reward conditioning is important for both stimulus-specific long-term memory (LTM) formation and extinction. It, however, remains unknown which components of memory formation Dnmts regulate (e.g., associative vs. non-associative) and in what context (e.g., varying training conditions). Here, we address these aspects in order to clarify the role of Dnmt-mediated DNA methylation in memory formation. We used a pharmacological Dnmt inhibitor and classical appetitive conditioning in the honeybee *Apis mellifera*, a well characterized model for classical conditioning. We quantified the effect of DNA methylation on naïve odor and sugar responses, and on responses following olfactory reward conditioning. We show that (1) Dnmts do not influence naïve odor or sugar responses, (2) Dnmts do not affect the learning of new stimuli, but (3) Dnmts influence odor-coding, i.e., ‘correct’ (stimulus-specific) LTM formation. Particularly, Dnmts reduce memory specificity when experience is low (one-trial training), and increase memory specificity when experience is high (multiple-trial training), generating an ecologically more useful response to learning. (4) In reversal learning conditions, Dnmts are involved in regulating both excitatory (re-acquisition) and inhibitory (forgetting) processes.

## Introduction

The ability of honey bees to learn and form memories has been described and investigated in depth for many years (Menzel, 2012). When bees forage they search for good food sources and memorize their features such as color, shape, and smell (Menzel, 2012). Bees show flower constancy during foraging (Chittka et al., 1999) and remember the features of a food source. On the other hand, it is also essential for bees to be able to re-evaluate their behavior, if a source no longer provides good quality food (Greggers and Menzel, 1993). Thus, extinction (i.e., forgetting) and re-acquisition are equally important. Furthermore, the environments bees encounter are variable, e.g., due to the slightly different smell of two flowers of the same species. Therefore, their ability to generalize stimuli belonging to the same category (e.g., type of flower) is as important as the ability to discriminate distinct stimuli (Shepard, 1987; Cheng, 2000). These different aspects and demands of foraging are reflected in bees cognitive capacities, and have been well documented in free-flying bees (Menzel, 2012).

Bee memory formation can be studied under controlled conditions with the proboscis extensions response (PER; Bitterman et al., 1983). In this assay, bees learn to associate an odor with a sugar reward, similar to the olfactory learning taking place when a bee collects nectar from a flower during foraging (Eisenhardt, 2014). Depending on the conditions used during training, the dynamics of memory formation differ; for example multiple, but not one, odor-sugar pairings cause a prolonged increase of protein kinase A (PKA; Hildebrandt and Muller, 1995; Müller, 2000). This suggests that different molecular pathways and dynamics may underlie memory formation depending on the training conditions.

Both few training trials and short time-intervals between training trials are associated with a reduced stimulus-specific memory, i.e., stronger generalization to novel stimuli (Perisse et al., 2009; Lefer et al., 2012). Generalization is the cognitive counterpart to perceptual discrimination (Cheng, 2000). It is dependent on stimulus similarity (e.g., different hues of blue, compared to yellow), but additionally requires a cognitive categorization of stimuli, which is experience dependent (Wright et al., 2008).

Relearning (e.g., extinction and re-acquisition during reversal learning) has been investigated with the PER assay as well (Eisenhardt and Menzel, 2007; Mota and Giurfa, 2010). Extinction describes the reduction in response to a previously learned stimulus when it is repeatedly presented without reward (Eisenhardt and Menzel, 2007). Reversal learning, on the other hand, consists in relearning the contingencies of stimuli (Mota and Giurfa, 2010). Extinction and reversal learning share common characteristics in that a previously formed association needs to be changed. They also both require processing in the mushroom bodies (MBs), a higher order brain center of bees (Devaud et al., 2007, 2015).

Epigenetic mechanisms are crucial for transcriptional regulation (Rothbart and Strahl, 2014; Schübeler, 2015). They comprise mechanisms which can tightly and subtly regulate transcription, thus being good candidates for regulating complex behaviors. In bees, epigenetic mechanisms – such as histone acetylation and DNA methylation – have been related to memory formation (Lockett et al., 2010; Biergans et al., 2012, 2015, 2016; Merschbaecher et al., 2012). Following olfactory reward conditioning proteins catalyzing DNA methylation (i.e., DNA methyltransferases, Dnmts) and demethylation (i.e., ten–eleven translocation methylcytosine dioxygenase, Tet) are upregulated and DNA methylation levels change in memory-associated genes (Biergans et al., 2015). In the presence of a Dnmt inhibitor global DNA methylation levels decrease in the brain and memory-associated genes are upregulated 24 h after training (Biergans et al., 2015). Furthermore, DNA methylation mediates associative plasticity in the neural network of the primary olfactory center and aids odor discrimination (Biergans et al., 2016). These studies support earlier behavioral data arguing for a role of DNA methylation in stimulus-specific LTM formation (Biergans et al., 2012) and extinction (Lockett et al., 2010).

Here, we investigated in detail the behavioral phenotypes these studies describe. Specifically, we assessed whether the observed effects after Dnmt inhibition are learning-dependent, replicable and robust. Furthermore, we re-analyzed all Dnmt inhibition experiments present to date to determine which functions of Dnmts during memory formation are best supported by the data.

## Results

### Dnmts Do Not Affect Odor or Sugar Perception in the Absence of Learning

**FIGURE 1:**
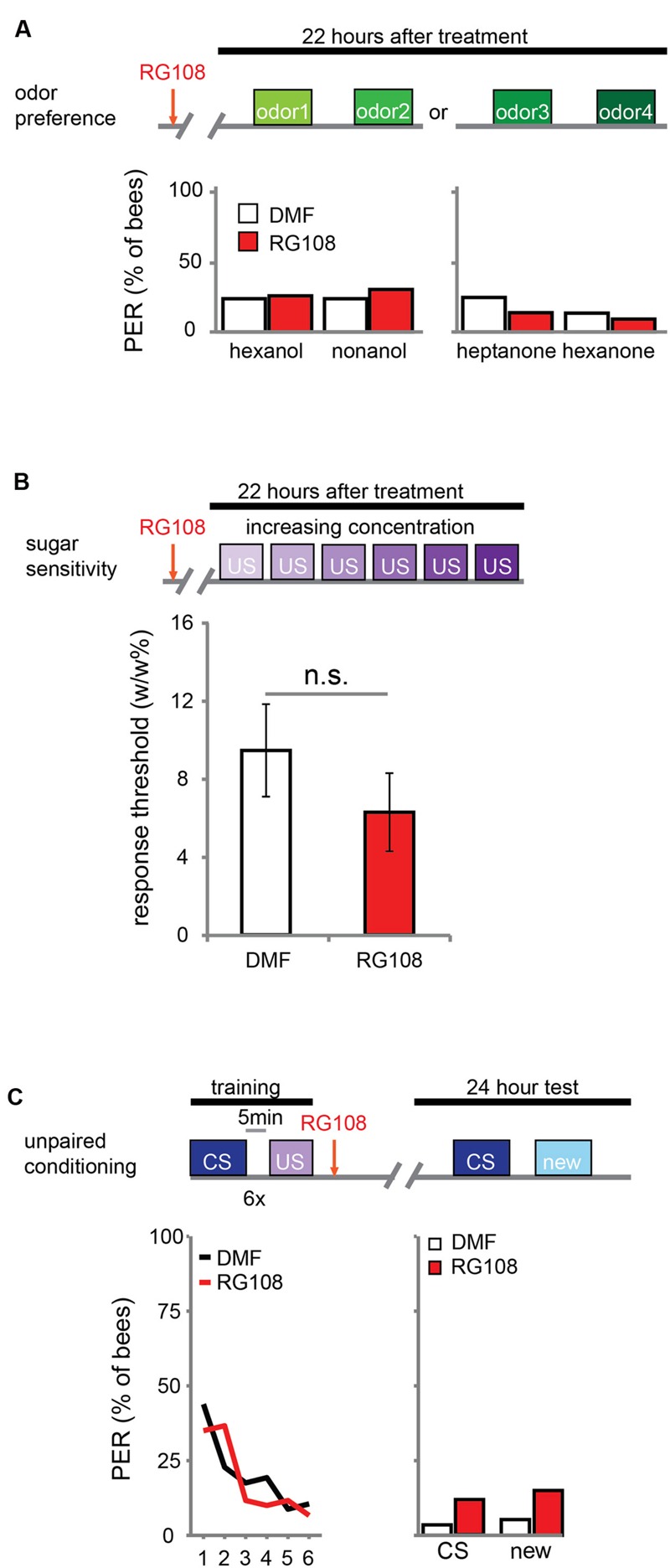
**DNA methyltransferases (Dnmts) do not affect odor or sugar perception in the absence of learning. (A)** The percentage of bees naïvely responding to all odors used in this study is shown. Bees were treated with 1 μl of the Dnmt inhibitor RG108 or the solvent DMF 22 h before the test, but no training took place. Two experiments: one with hexanol and nonanol [*n*(RG108) = 65, *n*(DMF) = 58], one with heptanone and hexanone [*n*(RG108) = 43, *n*(DMF) = 44]. Naïve odor responses were not different after RG108 treatment. **(B)** Bees were tested for their sugar responsiveness 22 h after RG108 treatment. Increasing concentrations of sugar water (0.1–30% w/w) were presented to their antennae. The response threshold is shown (mean +/- SEM). The response threshold was not different between RG108 and solvent treated bees [*n*(DMF) = 28, *n*(RG108) = 27]. **(C)** Although naïve odor responses were not affected by Dnmt inhibition the pre-exposure to the stimuli during training could be sufficient to change the response in the test even in the absence of learning. To control for a possible effect of pre-exposure, we trained bees with an unpaired paradigm [5 min between the CS (conditioned stimulus) and US (unconditioned stimulus)], treated them with RG108 or the solvent 2 h after training, and tested their response to the pre-exposed and a new odor 22 h later. The response did not differ between treatments [*n*(RG108) = 60, *n*(DMF) = 57]. n.s., not significant.

DNA methylation allows the animal to form a stimulus-specific LTM memory: when Dnmts are blocked, animals generalize more after learning. A possible explanation could be that Dnmt inhibition affects plasticity in stimulus perception rather than memory formation. Previous experiments already approached this hypothesis by treating bees with a Dnmt inhibitor 24 h before training. In these experiments acquisition, memory retention and generalization did not change (Lockett et al., 2010; Biergans et al., 2012). Here, we confirm that Dnmts do not affect perception in a context without learning. We treated bees with the Dnmt inhibitor RG108 or the solvent dimethylformamide (DMF) and tested their naïve odor preference (**Figure 1A**) and sugar sensitivity (**Figure 1B**) 22 h later. Additionally, we used unpaired conditioning where bees received odor and sugar separated by 5 min (**Figure 1C**). In this paradigm no memory is formed (Hellstern et al., 1998), but the cumulative stimuli experienced by the bee are the same as in appetitive learning studies. In all cases – odor preference, sugar sensitivity, and unpaired conditioning – there was no difference between Dnmt inhibitor treated and control bees (odor preference: glm, factor treatment: hexanol: *p* = 0.802; nonanol: *p* = 0.409; hexanone: *p* = 0.577; heptanone: *p* = 0.156; sugar sensitivity: glm, factor treatment: *p* = 0.314; unpaired conditioning: glm, factor treatment: CS: *p* = 0.118; new: *p* = 0.096). Thus, exposure to olfactory or gustatory stimuli in the absence of learning does not lead to DNA methylation changes that affect later odor responses. We conclude that the generalization effect observed after Dnmt inhibition is likely to be learning-dependent.

### Methylation Adjusts the Strength of Generalization Depending on the Training Conditions

**FIGURE 2:**
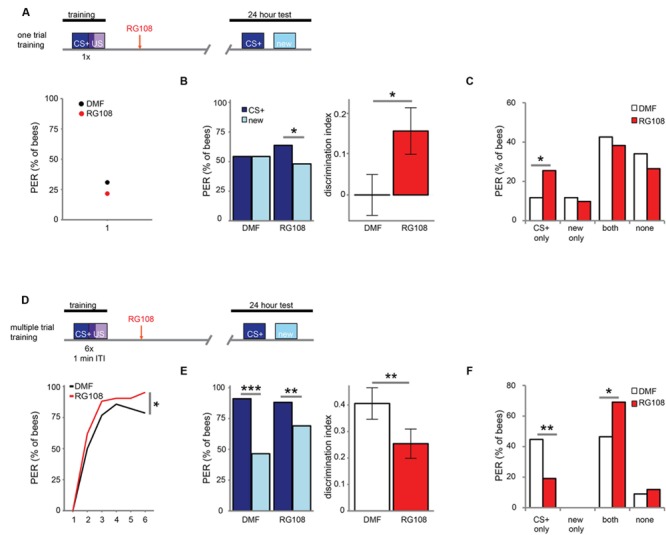
**DNA methyltransferases influence stimulus-specific memory bidirectionally. (A)** Bees were trained with one CS-US pairing **(D)** or six CS-US pairings with an inter-trial interval (ITI) of 1 min. Two hours after the training bees were treated with the Dnmt inhibitor RG108 or the solvent DMF (dimethylformamide) and tested for memory retention (CS+ response) and generalization (new response) after 24 h. **(B)** Solvent treated bees did not show stimulus-specific memory in the 24 h test following one CS-US pairing, but bees were able to discriminate between the CS+ and new odor after Dnmt inhibition [*n*(DMF) = 94, *n*(RG108) = 102]. **(C)** Bees were sorted into responding groups: bees responded more often only to the CS+ after Dnmt inhibition. **(D)** Bees were trained with six CS-US pairings. The group later treated with RG108 had a slightly better performance. **(E)** Bees’ stimulus-specific memory was impaired in RG108 treated bees after multiple trial training [*n*(DMF) = 56, *n*(RG108) = 42]. **(F)** Treated bees responded less to the CS+ only and more often to both odors. Note that the better performance of this group of bees (shown in **D**) would by its own lead to the opposite effect, thus making the effect of RG108 stronger. ^∗^*p*-value < 0.05; ^∗∗^*p*-value < 0.01; ^∗∗∗^*p*-value < 0.001.

Next, we investigated which training parameters influence how Dnmts affect stimulus-specific memory. We utilized two variations of PER conditioning which initiate distinct molecular pathways: single-trial learning and multiple-trial learning. First, we tested one trial training (i.e., only one odor-sugar pairing, **Figure 2A**). Control bees had a weak stimulus-specific memory after 24 h (**Figure 2B**). After Dnmt inhibition, however, bees formed a stimulus specific memory, successfully discriminating between the CS+ and a new odor (McNemar test, *p* = 0.011, effect size = 0.32). The number of bees responding correctly only to the CS+ increased after RG108 treatment (**Figure 2C**, χ^2^-test, *p* = 0.014, effect size = 0.37). Thus, after one-trial-training, Dnmt activity reduced odor selectivity in the memory trace.

Next, we tested multiple-trial (massed) training. We trained bees with six odor-sugar pairings, separated by 1 min each (**Figure 2D**). When Dnmts were inhibited, stimulus-specific memory formation was impaired and discriminatory power was significantly lower compared to control bees (**Figure 2E**, glm, *p* = 0.008, effect size = 0.56). Both the number of bees responding ‘correctly’ only to the CS+ was reduced (**Figure 2F**, χ^2^-test, *p* = 0.008, effect size = 0.56), and the number of bees responding ‘wrongly’ to both test odors was increased after Dnmt inhibition (**Figure 2F**, χ^2^-test, *p* = 0.026, effect size = 0.46). These data supplement previously published data with spaced multiple trial training (10 min intertrial interval), which also showed increased generalization when Dnmts were blocked (Biergans et al., 2012, 2015). Thus, while DNA methylation increases generalization after one trial learning, DNA methylation decreases generalization (increases odor recognition) in multiple-trial learning, leading to a more selective odor response (Biergans et al., 2016). This is an intriguing bi-directional effect of DNA methylation.

### Dnmts Regulate Both Extinction and Re-acquisition

**FIGURE 3:**
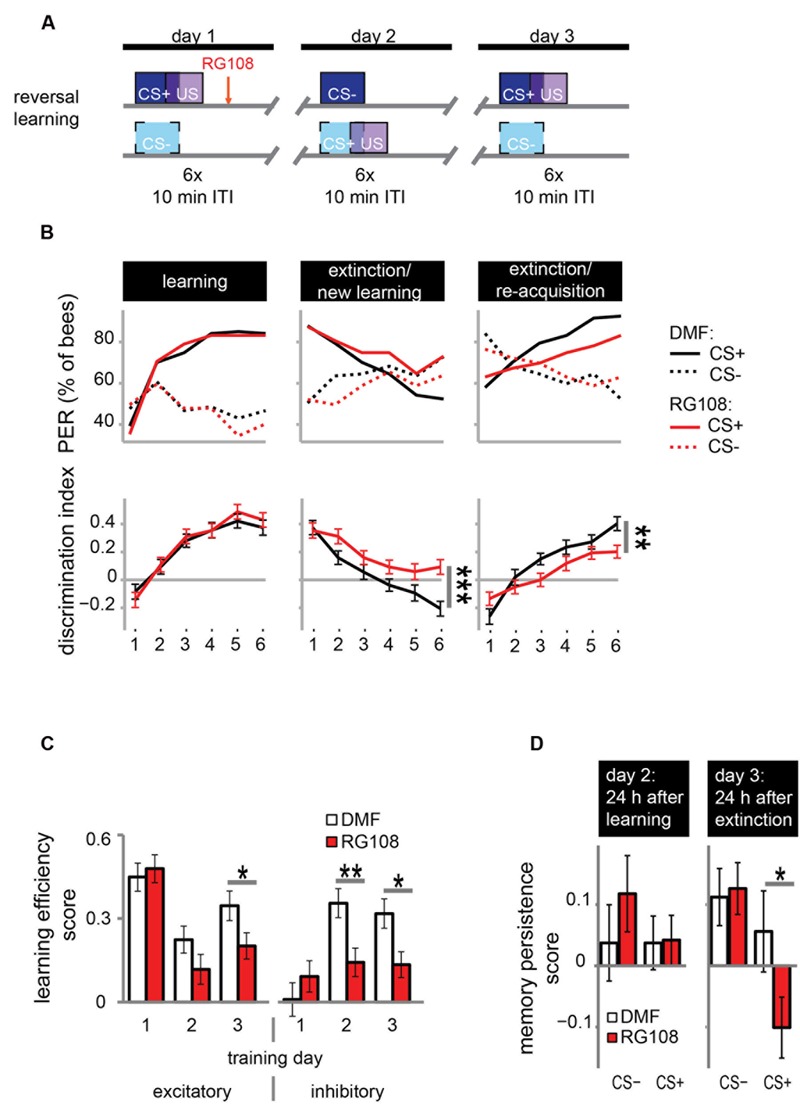
**DNA methyltransferases promote the relearning of previously learned stimuli. (A)** Bees were trained with a reversal training paradigm. **(B)** Solvent treated control bees responded correctly at the end of each training. RG108 treated bees, however, did not reverse the odor contingencies on day 2 (extinction/new learning) and performed significantly worse than control bees [*n*(DMF) = 107, *n*(RG108) = 119]. On day 3 (extinction/re-acquisition) they learned the reversal but did so significantly slower than control bees. **(C)** Learning efficiency scores (bees’ last minus its first training trial response; 0 = same response, 1 = successful learning, -1 = opposite response) were calculated for the excitatory and inhibitory components in each training. The excitatory component was impaired by RG108 treatment only on training day 3. The inhibitory component was impaired on training days 2 and 3. **(D)** Memory persistence scores (difference in a bees’ response between the last training trial and the first one 24 h later; 0 = same response, 1 = increase, -1 = decrease) were calculated for each period between trainings. RG108 treatment did not change the response to the CS+ or the CS- over the 24 h initial learning. During the second 24 h, however, RG108 treated bees changed their response to the unrewarded odor, but control bees did not. ^∗^*p*-value < 0.05; ^∗∗^*p*-value < 0.01; ^∗∗∗^*p*-value < 0.001.

DNA methyltransferases are also involved in extinction learning and memory (Lockett et al., 2010); i.e., the reduced response to a previously learned odor (‘*extinction*’) when the odor is repeatedly given without reward. We investigated whether Dnmts are also involved in relearning a previously forgotten odor (‘*re-acquisition*’)? We used a reversal learning paradigm. We trained bees three times, where each training was separated by 24 h (**Figure 3A**). Training was differential with one rewarded (CS+) and one unrewarded odor (CS-). The contingencies of odors were reversed every day, meaning that the odor which was rewarded on days 1 and 3 was unrewarded on day 2, and vice versa.

Control bees showed strong learning on day 1, extinction and new learning within four trials on day 2, and extinction and re-acquisition within three trials on the third day (black lines in **Figure 3B**). When treated with a Dnmt inhibitor (red lines in **Figure 3B**), however, bees were not able to learn the reversed contingencies of the odors on day 2, performing significantly worse than control bees (glm, *p* < 0.001, effect size = 0.54). Dnmt-inhibited bees were also significantly slower in learning during the extinction/re-acquisition phase on day 3 compared to control bees (glm, *p* = 0.005, effect size = 0.40).

Reversal learning consists of two components – an excitatory (i.e., increasing the response to the previously unrewarded odor) and an inhibitory component (i.e., decreasing the response to the previously rewarded odor; Mota and Giurfa, 2010). Thus, we analyzed these components separately in order to investigate whether Dnmts are involved in the regulation of either or both. We calculated the learning efficiency score for each training day and stimulus by subtracting the bees’ response in the first training trial from its response in the last (**Figure 3C**: 0 = no change in response, 1 = show learned response, -1 = show opposite effect) as described elsewhere (Mota and Giurfa, 2010). Dnmt inhibition caused a reduction of the inhibitory component on training days 2 and 3 and of the excitatory component on training day 3 (**Figure 3C**; glm, excitatory: day 2: *p* = 0.050, effect size = 0.27; inhibitory: day 2: *p* = 0.004, effect size = 0.39, day 3: *p* = 0.013, effect size = 0.35). Thus, both extinction (i.e., inhibitory component) and re-acquisition (i.e., excitatory component) relied on DNA methylation.

Next, we investigated whether the response after memory consolidation was also affected by the treatment induced impairments observed during training. We calculated a memory persistence score by subtracting the bees’ response in the last training trial from its response in the first training trial 24 h later (**Figure 3D**: 0 = same response 24 h after training, 1 = increased response, -1 decreased response). The bees’ responses at the end of the learning phase on day 1 were largely maintained at the beginning of the day 2 (**Figure 3D** ‘24 h after learning’). Twenty-four hours following the extinction/new learning phase, however, bees’ memory retention was improved for the initial CS+. RG108 treated bees also maintained the response to the initially learned odor at the beginning of day 2, but showed reduced responses to that odor at the beginning of day 3 (**Figure 3D** ‘24 h after extinction,’ glm, *p* = 0.032, effect size = 0.26). This suggests that the impairment of the inhibitory learning component during the second training day is compensated during memory consolidation between days 2 and 3.

**FIGURE 4:**
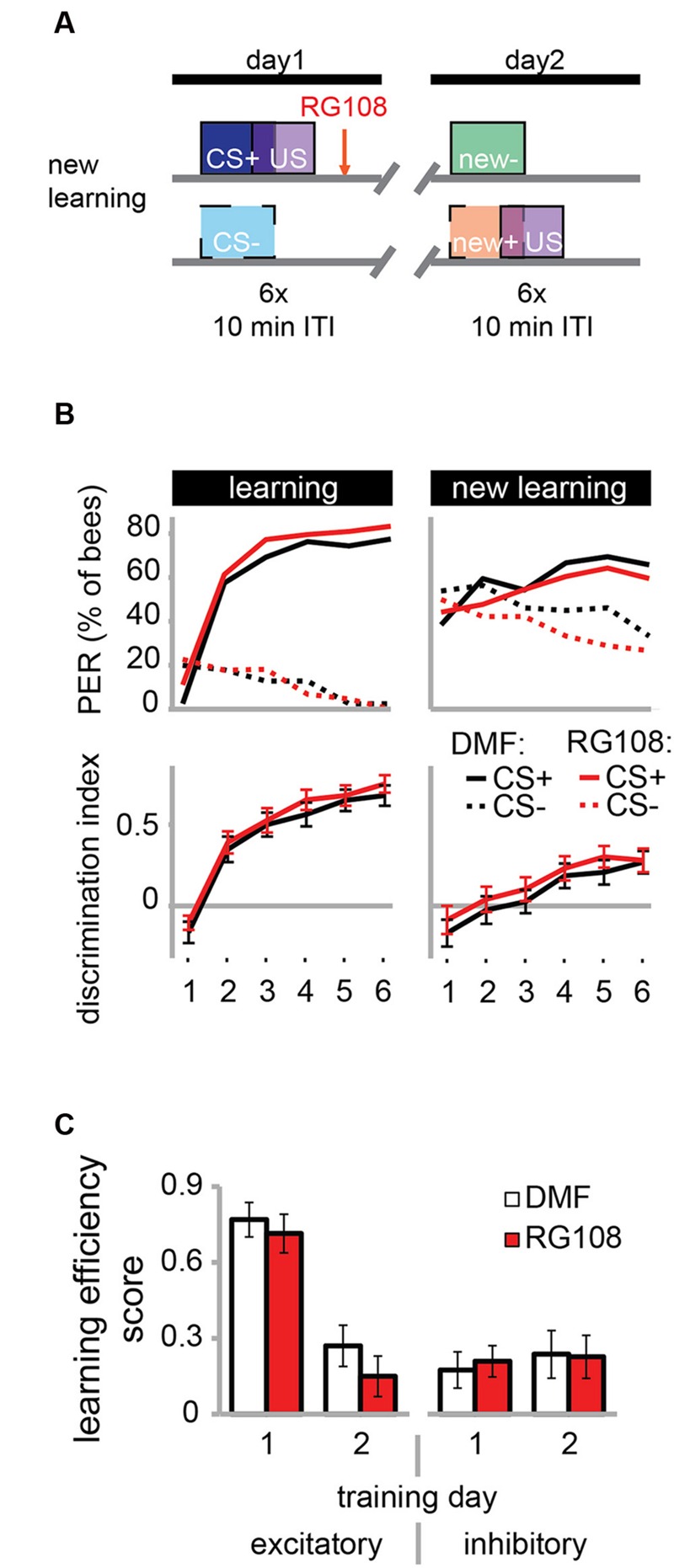
**DNA methyltransferases do not regulate the learning of new odors. (A)** To confirm that Dnmts are necessary for efficient relearning of previously learned stimuli, but not for acquisition in general, we trained bees with a differential conditioning paradigm as in **Figure 3**, but trained them with two new odors on day 2. **(B)** Bees learned to discriminate the two new odors with and without active Dnmts. **(C)** Neither excitatory nor inhibitory components of learning were impaired by Dnmt inhibition on the second training day. *n*(DMF) = 39, *n*(RG108) = 44.

The necessity of active Dnmts during reversal learning could indicate that Dnmts are either important for the re-learning of previously learned stimuli or for the ability to learn in general. So far there is more evidence for the first hypothesis, as Dnmts are not necessary during acquisition in naïve bees (Lockett et al., 2010; Biergans et al., 2012). In the paradigm used in **Figure 3**, however, bees learned in the context of previous training, creating a situation where the effect of learning ability or re-learning ability cannot be separated. Therefore, we modified the protocol as follows: We trained bees as described before with a differential training paradigm including a rewarded (CS+) and an unrewarded odor (CS-). On day 2, however – instead of re-training with the previously used odors – we trained bees with two new odors (**Figure 4A**). We found that both the solvent treated control bees and RG108 treated bees were able to learn to discriminate the new odors during the second training day (**Figure 4B**). None of the learning components was affected by Dnmt inhibition (**Figure 4C**). This confirms that Dnmt activity was not important for acquisition in general, but it was important specifically for the relearning of previously learned stimuli.

### A Role of Dnmts in ‘Correct’ LTM Formation, Reversal Learning, and Extinction MTM Is Supported by the Experimental Data to Date

**FIGURE 5:**
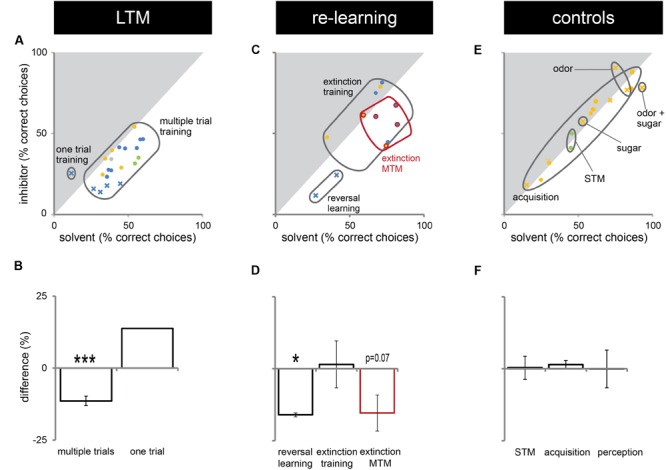
**Correct long-term memory (LTM) formation and relearning are both facilitated by Dnmts.** All PER experiments shown in this study, published previously (Lockett et al., 2010; Biergans et al., 2012, 2015) or performed additionally (summarized in **Supplementary Table S1**), were re-analyzed in order to gain an overview of which roles of Dnmts are best supported by the data. The % of bees responding ‘correctly’ was calculated for the treatment and control groups. What a ‘correct’ response was differed between experiments (**Table 1**). **(A,C,E)** The % of correct responses for the solvent and inhibitor treated groups are plotted against each other. Each mark represents one experiment (inhibitor: X = RG108, O = zebularine; treatment time-point: yellow = before; green = before + after; blue = after training). **(A)** Solvent treated bees responded ‘correctly’ more often than inhibitor treated bees in most experiments testing LTM retention after multiple trial olfactory reward conditioning. **(B)** Pooling the difference between the ‘correct’ responses in inhibitor and solvent treated bees of all experiments shows a significant effect of Dnmt activity (*n* = 21). **(C,D)** Reversal learning (*n* = 2) and extinction MTM (*n* = 5) were most consistently impaired by Dnmt inhibition, whereas the results for extinction learning (*n* = 5) were inconsistent. **(E,F)** Control experiments so far tested the effect of Dnmt inhibition on STM, acquisition, and perception. There was no consistent effect on either [*n*(STM) = 2, *n*(Acquisition) = 10, *n*(Perception) = 4]. ^∗^*p*-value < 0.05; ^∗∗∗^*p*-value < 0.001.

In this study, we presented experiments showing that Dnmts regulate stimulus-specific LTM and re-learning, but do not affect stimulus perception or acquisition of new stimuli. In order to compare these results to and imbed them with the body of data available in the literature so far, we performed a meta-analysis. We aggregated available published data (Lockett et al., 2010; Biergans et al., 2012, 2015), unpublished data (summarized in **Supplementary Table S1**) and all experiments shown in this study. We formed three categories: (1) experiments testing LTM (**Figures 5A,B**), (2) experiments testing re-learning (**Figures 5C,D**), and (3) control experiments (**Figures 5E,F**). All experiments used odor reward conditioning and PER as a behavioral read-out. They differed, however, in the training paradigm used (i.e., absolute or differential) and the stimuli tested (CS+, CS-, new odor, sugar). We calculated the % of bees responding ‘correctly’ in the inhibitor treated group (reduced Dnmt activity) of each experiment and plotted this value against the % of bees responding correctly in the solvent treated group (normal Dnmt activity, **Figures 5A,C,E**). The scores differed across the training/test paradigm used (summarized in: **Table 1**). The individual experiments further differed in the inhibitor used [i.e., RG108 (**X**) or Zebularine (**O**)] and the treatment timepoint [i.e., before (yellow: **O**), before + after (green: **O**), or after (blue: **O,X**) training]. In **Figure 5A**, a point on the diagonal indicates an experiment where Dnmt activity does not affect ‘correct’ LTM formation; points below the diagonal (lower-right) indicate experiments where Dnmt activity positively contributed to ‘correct’ memory performance.

**Table 1:** Evaluation of correct behavioral responses in the analyzed data sets.

Training/Test	CS+	CS-	New odor	Sugar
Differential training/test	PER	No PER	X	X
Absolute training/test	PER	X	No PER	X
Unpaired training/test	No PER	X	No PER	X
Extinction learning/MTM	No PER	X	X	X
Naïve odor test	X	X	No PER	X
Naïve sugar test	X	X	X	PER

*Depending on the training paradigm and test used a ‘correct’ response differed. The trained and tested stimuli are shown, and what comprised a ‘correct’ response in the specified training/test. ‘X’ indicates that this stimulus was not present in the specified training/test.*

Most LTM experiments showed a reduction of ‘correct’ responses after Dnmt inhibition (points below the diagonal in **Figures 5A,B**, one sample *t*-test, *p* < 0.001, effect size = 1.516). Thus, the data suggest a role of Dnmts in facilitating ‘correct’ LTM formation with an average reduction of 11% in ‘correct’ responses when Dnmts are pharmacologically inhibited. Data also show a role of Dnmts in extinction mid-term memory (MTM, **Figures 5C,D**, red circles, one sample *t*-test, *p* = 0.070) and reversal learning (**Figures 5C,D**, one sample *t*-test, *p* = 0.028, effect size = 16.052). For extinction learning, however, data are inconsistent, with some experiments showing a reduction in extinction and some an increase after Dnmt inhibition (**Figures 5C,D**, one sample *t*-test, *p* = 0.865). The difference may be due to the treatment time-point. In order to control for a potential effect of Dnmts on acquisition or stimulus perception, all control experiments performed were also pooled (**Figures 5E,F**). There was no consistent effect on short-term memory, acquisition, or perception.

## Discussion

Long-term memory (LTM) needs neural networks that are modified in a stable way over several days up to a life time. Epigenetic modifications of the genome in neurons have been shown to contribute to these LTM traces (Zovkic et al., 2013). However, every memory consists of several phases (over time), content (e.g., stimulus specificity, associative strength), and involves distinct neural networks across the brain. Therefore, it is important to dissect the exact role of a particular molecular mechanism in order to build a complete picture of how memory functions in a living animal. Here, we show that DNA methylation fulfills very specific roles in a honeybee olfactory reward memory paradigm. Specifically, we show that Dnmts regulated stimulus-specific LTM robustly under differing training parameters, whereas the directionality of the regulation depended on the trial number during training (**Figure 2**). Additionally, we show that Dnmts regulated extinction and thus the inhibitory component of re-learning, and also the excitatory component in a reversal learning paradigm (**Figure 3**). Furthermore, we re-evaluated the evidence available to date focusing on the role of Dnmts in olfactory reward conditioning and found that Dnmts consistently play a role in ‘correct’ LTM formation (e.g., stimulus-specific memory formation), reversal learning and extinction MTM, whereas its role in extinction learning remains unresolved at this point (**Figure 5**).

DNA methyltransferase inhibition did not affect naïve odor and sugar responses or odor responses after unpaired training (i.e., without memory formation). These results confirm earlier studies showing that acquisition is not affected by Dnmt inhibition 24 h before (Lockett et al., 2010; Biergans et al., 2012), which argues against an effect of Dnmts on naïve stimulus perception. This suggests that Dnmt activity (and/or expression) is induced by the coincident occurrence of conditioned stimulus (CS) and unconditioned stimulus (US) during learning. Indeed, it has recently been shown that Dnmts and also the demethylation protein Tet are upregulated after olfactory reward conditioning (Biergans et al., 2015).

DNA methylation via Dnmts regulates stimulus-specific LTM after olfactory reward conditioning (Biergans et al., 2012, 2015). The directionality of this regulation, however, depended on the number of training trials, and was independent of the inter-trial interval (**Figure 2**). After one odor-sugar pairing control bees formed a weak stimulus-specific memory, which confirms earlier studies (Perisse et al., 2009; Lefer et al., 2012). Following Dnmt inhibition, however, bees were able to discriminate between the trained and a new odor. Thus, Dnmt-dependent mechanisms seem to increase generalization after one trial training and decrease generalization after multiple trial training. Without the activity of Dnmts, generalization is comparable in the two situations. Thus, we can speculate that the adaptive role of Dnmts in regulating memory is the following: a single odor-sugar pairing is not sufficient to predict that a particular odor is rewarded, and Dnmt activity therefore weakens the odor identity related information in the memory trace. Repeated pairings, on the other side, indicate reliable odor-information, and methylation increases odor-specific memory information. This differential effect may be based on differing molecular pathways. Multiple trial training induces long-lasting PKA and PKC activity and is counter-acted by protein degradation, whereas one trial training is not (Hildebrandt and Muller, 1995; Grünbaum and Müller, 1998; Müller, 2000; Felsenberg et al., 2012). At this point there is not enough information about how Dnmts may regulate stimulus-specific memory bidirectionally. We, however, discuss two tentative possibilities: (1) Dnmts have been shown to have demethylating activity, in addition to their predominant methylating activity (Chen et al., 2013). This reversal in function is related to Ca^2+^ levels. Differences in stimulus-specific memory formation between one and multiple trial training may be related to differing Ca^2+^ levels after training (Perisse et al., 2009). Thus, different Ca^2+^ levels present in neurons after training may regulate whether Dnmts are active as methylase or as demethylase. It has to be noted though that Dnmt demethylase activity has only been described under specific conditions *in vitro* yet. (2) Another possibility is that the different molecular pathways triggered after one and multiple trial training (Hildebrandt and Muller, 1995; Müller, 2000; Perisse et al., 2009; Felsenberg et al., 2012) cause Dnmts to target different genes. Similarly, histone modifications also follow different dynamics after one and multiple trial training (Merschbaecher et al., 2012). Thus, Dnmts and related up- and downstream processes might fine-tune memory formation depending on the environmental information available and thus allow for maximally beneficial adjustments in an animals’ behavior.

DNA methyltransferases also regulate extinction in bees (Lockett et al., 2010). Extinction is a form of re-learning during which bees need to re-evaluate a previously rewarded stimulus as being not rewarded any more. Compared to this, during reversal learning, bees have to re-evaluate two stimuli simultaneously, with one being rewarded and the other one not. Evidence from both extinction and reversal learning studies favors the idea that ‘positive’ and ‘negative’ memories of a stimulus are present in parallel (Stollhoff et al., 2005; Mota and Giurfa, 2010). Our data suggests that both the inhibitory component of reversal learning (i.e., extinction) and the excitatory component (i.e., re-acquisition) involve Dnmts. Dnmts may regulate these processes in two ways: (1) Dnmts could affect the balance between opposing memory traces for a stimulus. This could cause a behavioral dominance of the most recent association learned over older memories during training. (2) Dnmts could be involved in de-constructing the older memory trace. Further experiments investigating extinction and reversal learning and the underlying molecular mechanisms are needed to gain insight into what the specific function of Dnmts is here. Interestingly, even though relearning was impaired during the training, memory recall 24 h after training was not, whereas subsequent relearning was again impaired. Thus, it seems as if Dnmts regulate pathways needed during relearning, but that the memory is consolidated correctly without the need for DNA methylation. Notably, only the relearning of previously learned stimuli was impaired when Dnmts are inhibited, but not bees’ general ability to learn. This suggests that Dnmts set methylation marks only in those neurons active during training (e.g., neurons responding to a particular odor) and thus potentially create a memory trace on the level of the chromatin mirroring the activity of that neuron over time.

Comparable studies in mammals found that DNA methylation is involved in extinction memory (Rudenko et al., 2013; Morris et al., 2014), similarly to what has been shown in honey bees (Lockett et al., 2010). On the other hand, DNA methylation affects CS+ memory strength in mammals (Zovkic et al., 2013), but not in honey bees (Lockett et al., 2010; Biergans et al., 2012, 2015, 2016). A potential role of DNA methylation in regulating memory specificity and re-acquisition has not been assessed in mammals so far. Histone deacetylation has, however, been shown to affect memory specificity after auditory conditioning in mice (Bieszczad et al., 2015), suggesting that epigenetic regulation of memory specificity might be conserved across animals.

With ‘correct’ LTM formation and relearning, we now know that Dnmts are involved in two distinct groups of behavioral readouts after olfactory reward conditioning in bees (**Figure 4**). Further studies will need to investigate how exactly Dnmts regulate these behaviors and whether the same Dnmt targeted genes affect both, or whether distinct sets of genes are required for each. Furthermore, it will be crucial to investigate the role Dnmts play in learning paradigms utilizing different CS (e.g., visual stimuli) and US (e.g., punishment). This will reveal which neuronal networks and brain centers are involved and whether Dnmts regulate the same processes independent of the sensory modalities utilized during training.

## Materials and Methods

### Odor Reward Conditioning and Memory Retention Test

Experiments were performed either at the University of Queensland (Brisbane, QLD, Australia) or the University of Konstanz (Konstanz, Germany). Honey bees (*Apis mellifera*) were caught outside the hive and put on ice until they were immobilized. Bees were harnessed in plastic tubes so that they could only move their head, but with their thorax still accessible. They were fed until satiation and kept overnight in a humid plastic box, or an incubator depending on where the experiment was performed. The next day bees were trained using an appetitive olfactory training paradigm. The exact training parameters were different for each experiment (summarized in **Table 2**). In all experiments the odor (CS) was presented for 4 s and sugar reward (1 mM sugar water, US) for 3 s. Odors (all Sigma–Aldrich) were dissolved in hexane for the experiments performed in Australia and in mineral oil (in all cases diluted 10^2^) for those performed in Germany.

**Table 2:** Overview over training parameters.

Figures	NoT	ITI (min)	ISI (s)	Location
1a	NA	NA	NA	Germany
1b	NA	NA	NA	Australia
1c	6	10	300	Germany
2a	1	NA	2	Australia
2b	6	1	2	Germany
3	6 (differential)	10	2	Australia
4	6 (differential)	10	2	Germany

*For each experiment presented here the number of trials (NoT), inter-trial interval (ITI), inter stimulus interval (ISI) and location, where the experiment was performed, is shown.*

During each experiment bees experienced a constant air-flow in order to avoid mechanical stimulation at odor onset. Twenty-four hours after training bees were tested for memory retention by presenting them with the CS+ (trained odor) and a new odor, which was not present during the training, in randomized order. 1-hexanol and 1-nonanol were alternated as CS+, CS- or new odor, respectively. Additionally, for the control experiments shown in **Figure 4** 1-heptanone and 1-hexanone were used on the second day for training.

### Control Experiments

During the control experiments investigating whether Dnmt inhibition affects stimulus perception (**Figure 1**), bees did not receive olfactory appetitive training. Instead, bees were tested for ‘naïve’ odor or sugar responses 22 h after treatment – equivalent to the time trained bees were tested after treatment. For the odor preference test, bees were tested for their spontaneous proboscis extension response to all four odors used here in two separate experiments. 1-hexanol and 1-nonanol were always tested together and their order was alternated across bees (the same for 1-hexanone and 1-heptanone). For their sugar response bees were tested with increasing concentrations of sugar water (0.1, 0.3, 1, 3, 10, 30% w/w). Bees’ antennae were touched with a tooth pick soaked in sugar water and it was recorded whether or not bees extended their proboscis in response. The lowest concentration a bee responded to (response threshold) was compared between treatments. Before and after the test, as well as after each individual sugar concentration, bees were tested for their response to water. Bees responding to water more than twice or not responding to the highest sugar concentration were discarded from the experiment.

### Treatment

Two hours after training and 22 h before the control experiments bees were treated with 1 μl of the Dnmt inhibitor (RG108, 2 mM in DMF, Sigma–Aldrich) or the solvent DMF topically applied on the back of the thorax as described elsewhere (Lockett et al., 2010; Biergans et al., 2012, 2015). RG108 was chosen based on a comparative study involving the two Dnmt inhibitors RG108 and zebularine (Biergans et al., 2015). That study had shown the two substances to be comparable, with slightly stronger effects for RG108. Similarly, the treatment time-point and concentrations corresponded to that of earlier studies (Lockett et al., 2010; Biergans et al., 2012, 2015, 2016).

### Data Analysis

For all experiments the % of bees responding to the odors in the test and training was calculated. Furthermore, a discrimination index was calculated. The response of each individual bee to the new odor or CS- was subtracted from its response to the CS+. All data were analyzed using generalized linear models, if treatment groups were compared. To compare the response to the CS+ and new odor within one treatment group, a McNemar test was used. The McNemar test is suitable to compare binary, paired data.

For the meta-analysis, we gathered all honeybee data investigating the role of Dnmts in memory formation, including published data (Lockett et al., 2010; Biergans et al., 2012, 2015), data presented in this study and unpublished data. An overview over all data sets used for the meta-analysis is shown in **Supplementary Table S1**. We calculated the number of ‘correct’ responses within each experiment and experimental group (**Table 1**).

Using this method, we were able to compare data obtained by different training paradigms and assess the overall evidence for the effect of Dnmt inhibition. To quantify the level of agreement between different studies, we calculated the difference in the correct responses of inhibitor and solvent groups for each experiment and pooled them. A two-sided one-sample *t*-test was used to test whether the effect shown in those studies is reliably different from 0. The effect size [Cohen’s D (Navarro, 2015)] was calculated for all effects reaching the 0.05 significance level. As a guideline effect sizes below 0.2 are described as negligible, between 0.2 and 0.5 as small, between 0.5 and 0.8 as medium and above 0.8 as large (Cohen, 1992). The effect size can be used as an estimate of the real difference between the tested groups. All analyses were performed using custom written R-scripts using R-version 3.2.1 (R Core Team, 2015).

## Author Contributions

SB, CC, JR, and CG conceived and designed the study. SB performed the experiments. SB analyzed the experiments. SB wrote the paper. CC and CG edited the paper.

## Conflict of Interest Statement

The authors declare that the research was conducted in the absence of any commercial or financial relationships that could be construed as a potential conflict of interest.

## Supplementary Material

TABLE S1 **Summary of all Dnmt inhibition studies to date.** The table includes the following information: experimental IDs and the training paradigm used. The Dnmt inhibitor (Inhib.) used which was either zebularine or RG108 and the solvent DMF as control. In all cases the inhibitor was applied topically onto the thorax except if an “i” behind the inhibitor’s name indicates it was injected into the thorax. Odors were either vanilla in sugar water (Vsu), limonin in salt water (Lsa), 1-hexanol (HexL), 1-nonanol (NonL), 1-hexanone (HexN) or 1-heptanone (1-HepN). Test and treatment timepoints are stated in hours relative to the training. The number of bees (NoB) in each group is indicated and the number of bees responding correctly (RR) or wrongly (WR) as defined in **Table 1**. The percent of bees responding correctly is indicated C.R. (%). Furthermore the reference (Ref) is given for each previously published dataset: 1 – Lockett et al., 2010, 2 – Biergans et al., 2012, 3 – Biergans et al., 2015.
